# Influenza A virus infection in zebrafish recapitulates mammalian infection and sensitivity to anti-influenza drug treatment

**DOI:** 10.1242/dmm.014746

**Published:** 2014-09-04

**Authors:** Kristin A. Gabor, Michelle F. Goody, Walter K. Mowel, Meghan E. Breitbach, Remi L. Gratacap, P. Eckhard Witten, Carol H. Kim

**Affiliations:** 1 Graduate School of Biomedical Sciences and Engineering, University of Maine, Orono, ME 04469, USA.; 2 Department of Molecular and Biomedical Sciences, University of Maine, Orono, ME 04469, USA.; 3 Department of Biology, Ledeganckstraat 35, B-9000 Ghent, Belgium.

**Keywords:** Influenza, Zebrafish, Virus, Innate immunity

## Abstract

Seasonal influenza virus infections cause annual epidemics and sporadic pandemics. These present a global health concern, resulting in substantial morbidity, mortality and economic burdens. Prevention and treatment of influenza illness is difficult due to the high mutation rate of the virus, the emergence of new virus strains and increasing antiviral resistance. Animal models of influenza infection are crucial to our gaining a better understanding of the pathogenesis of and host response to influenza infection, and for screening antiviral compounds. However, the current animal models used for influenza research are not amenable to visualization of host-pathogen interactions or high-throughput drug screening. The zebrafish is widely recognized as a valuable model system for infectious disease research and therapeutic drug testing. Here, we describe a zebrafish model for human influenza A virus (IAV) infection and show that zebrafish embryos are susceptible to challenge with both influenza A strains APR8 and X-31 (Aichi). Influenza-infected zebrafish show an increase in viral burden and mortality over time. The expression of innate antiviral genes, the gross pathology and the histopathology in infected zebrafish recapitulate clinical symptoms of influenza infections in humans. This is the first time that zebrafish embryos have been infected with a fluorescent IAV in order to visualize infection in a live vertebrate host, revealing a pattern of vascular endothelial infection. Treatment of infected zebrafish with a known anti-influenza compound, Zanamivir, reduced mortality and the expression of a fluorescent viral gene product, demonstrating the validity of this model to screen for potential antiviral drugs. The zebrafish model system has provided invaluable insights into host-pathogen interactions for a range of infectious diseases. Here, we demonstrate a novel use of this species for IAV research. This model has great potential to advance our understanding of influenza infection and the associated host innate immune response.

## INTRODUCTION

Influenza virus infections lead to substantial illness, mortality and social disruption worldwide. In the United States alone, seasonal influenza is associated with an estimated 95,000–172,000 hospitalizations and 21,000–41,000 deaths, costing billions of dollars in healthcare expenses annually (Lowen et al., 2006). Influenza infections initially occur in epithelial cells of the respiratory tract and can become systemic, potentially resulting in multi-organ failure and death. Influenza A virus (IAV) is the most extensively studied type of influenza virus because of the threat of a pandemic outbreak. New strains of IAV with altered pathogenicity and/or antiviral resistance emerge with high frequency due to the segmented, single-stranded RNA genome of IAV, which allows for both antigenic drift and antigenic shift (Medina and García-Sastre, 2011). Vaccines against specific viral antigens are used in an attempt to provide hosts with immunity to IAV. However, the constantly changing genome of influenza A viruses makes it problematic to prevent or treat influenza-mediated diseases through targeting the virus itself. A better understanding of the host’s innate antiviral response to IAV could elucidate new targets for antiviral medicines or adjuvant therapies.

Animal models of influenza infection are necessary to study influenza pathogenesis and the host response to IAV. Several models of IAV infection exist, each with unique advantages and limitations. The hallmarks for such models are: susceptibility to influenza infection, support of viral replication and recapitulation of clinical symptoms of human disease. The current mammalian species used for IAV research, such as mice, ferrets or macaques (Bouvier and Lowen, 2010), are invaluable for studies on transmission, adaptive immunity and vaccine development. The development of a model in which large-scale chemical and genetic screens can be conducted will complement existing avenues of IAV research. We sought to use the zebrafish (*Danio rerio*) as an alternative vertebrate animal model for influenza infection studies because early-life stage zebrafish are highly amenable to genetic manipulation, transgenesis and high-throughput drug testing. An additional advantage of this model system is that zebrafish rely solely on their innate immune response for the first 4–6 weeks of development (Lam et al., 2004; Trede et al., 2004; Meeker and Trede, 2008; Kanther and Rawls, 2010). Because a robust innate immune response potentiates a subsequent heightened adaptive immune response, zebrafish embryos provide an opportunity to study the initial infection events to which the innate immune system responds, independently of adaptive immunity. New targets for antiviral drugs or adjuvant therapies could come from zebrafish studies of the host’s innate immune response to IAV.

Zebrafish models of infectious diseases are well established. Bacterial, fungal and viral infections have been characterized in zebrafish (reviewed in Trede et al., 2004; van der Sar et al., 2004; Sullivan and Kim, 2008; Tobin and Ramakrishnan, 2008; Lieschke and Trede, 2009; Tobin et al., 2012). To date, studies of viral pathogens that can infect and cause disease in zebrafish have been limited primarily to fish-specific viruses (LaPatra et al., 2000; Sanders et al., 2003; Phelan et al., 2005; Novoa et al., 2006; Lu et al., 2008; Xu et al., 2008; Encinas et al., 2010; Gabor et al., 2013). Recent studies have now shown that zebrafish can be infected with mammalian and/or human viruses. Adult zebrafish have been infected with herpes simplex virus type 1 (Burgos et al., 2008), and zebrafish larvae have been shown to support Chikungunya virus infection (Palha et al., 2013).

**TRANSLATIONAL IMPACT**

**Clinical Issue**

Influenza A virus (IAV) is a pathogen that poses a significant risk to human health. The emergence of new strains of IAV with altered virulence and increasing antiviral resistance are major clinical concerns. The high frequency of occurrence, the potential for global pandemics and the economic burden of influenza infection warrant the need for a better understanding of influenza pathogenesis and the host response so that new antiviral medicines can be developed. Model organisms are crucial for testing the safety and efficacy of potential therapeutics, but to date there has been no animal model of influenza infection in which high-throughput chemical screens could be conducted.

**Results**

In this study, it is demonstrated that zebrafish embryos can be infected with human isolates of IAV. A model of influenza viremia was generated by injecting IAV into the bloodstream of zebrafish embryos at 2 days post fertilization (dpf). An influenza infection of the respiratory tract was recapitulated by injecting IAV into the swimbladder of 5-dpf larvae because the swimbladder is considered to be anatomically and functionally analogous to the mammalian lung. Upon challenge with IAV, survival was reduced compared with uninfected controls, and viral burden was detected in infected zebrafish. IAV-infected zebrafish displayed heightened innate immune responses, as indicated by increased expression of innate antiviral genes (*ifnφ1* and *mxa*). Gross pathology and histopathology revealed symptoms similar to human influenza infections, including edema and tissue destruction. The use of a fluorescent reporter strain of IAV enabled visualization of IAV infection *in vivo*, demonstrating the potential for studies of host-pathogen interactions in real time. Finally, treatment of infected zebrafish with an established IAV antiviral ameliorated mortality, as well as viral replication and burden (i.e. GFP expression of the fluorescent reporter strain of IAV).

**Implications and future directions**

The results presented in this work show that the mechanisms of influenza infection are similar in humans and zebrafish, thus validating the therapeutic efficacy of this model. The advantages of zebrafish embryos were exploited to visualize IAV infection *in vivo* and to rapidly confirm the antiviral activity of a known anti-influenza compound. The use of the zebrafish embryo model for infectious disease studies provides the opportunity for future studies involving 4D imaging, genetic screening and high-throughput drug discovery. These future studies have the potential to enhance our understanding of influenza infection and of the establishment of the antiviral state. Ultimately, this new animal model of IAV infection could lead to the identification of drug targets for therapeutic intervention of IAV in humans.

Here, the zebrafish is established as a useful animal model for studying human IAV infection. The sialic acid linkages known to bind to human IAV were identified in zebrafish embryos, and infection studies using two strains of IAV demonstrated that viral burden and mortality increased over time. Cytokine profiling revealed that an antiviral state was induced in IAV-infected zebrafish, as it is in humans. Several aspects of gross pathologic and histopathological findings, such as edema and tissue necrosis, were similar between infected zebrafish and human IAV infections. A genetically modified fluorescent reporter strain of IAV (NS1-GFP) (Manicassamy et al., 2010) was utilized to visualize infection in a transparent vertebrate host. Finally, an antiviral compound used to treat IAV infection in humans reduced the mortality and observed viral burden in IAV-infected zebrafish. Herein, the first studies establishing the zebrafish as a model for human influenza infection are presented, and it is shown that IAV infection proceeds and can be resolved through similar mechanisms in zebrafish and humans.

## RESULTS

### α-2,6-linked sialic acids are present in zebrafish embryos

In order for cellular entry to occur, the IAV hemagglutinin protein binds to host cell surface receptors containing terminal sialic acid residues (Gottschalk, 1959). Human IAV isolates have a higher affinity for terminal α-2,6-linked sialic acid-containing receptors than other sialic acid linkages (Skehel and Wiley, 2000). It has been demonstrated that zebrafish synthesize a variety of sialylated glycoconjugates during the early stages of development and α-2,6-linked sialic acids have been predicted to occur in zebrafish embryos (Marx et al., 2001; Chang et al., 2010; Dehnert et al., 2012; Langhauser et al., 2012; Schaper et al., 2012). In order to confirm the presence of α-2,6-linked sialic acids, the specific sialic acid linkages in wild-type zebrafish embryos at 48 hours post fertilization (hpf), were identified using anion-exchange chromatography. Both N-acetylneuraminic acid (NANA) and N-glycolylneuraminic acid (NGNA) were detected in zebrafish embryos (supplementary material Table S1).

The abundance of specific sialic acid linkages (i.e. α-2,6 and α-2,3) was quantified, and Table 1 shows the detection of α-2,6-linked sialic acids in zebrafish embryos (see also supplementary material Fig. S1). Analysis of the sialic acids revealed no detectable α-2,3-linked sialic acids in the same embryo samples (Table 1). These results show that sialic acids with α-2,6 linkages are present in zebrafish embryos and that human IAV isolates should be able to attach to, and potentially enter, zebrafish embryo cells.

**Table 1.**
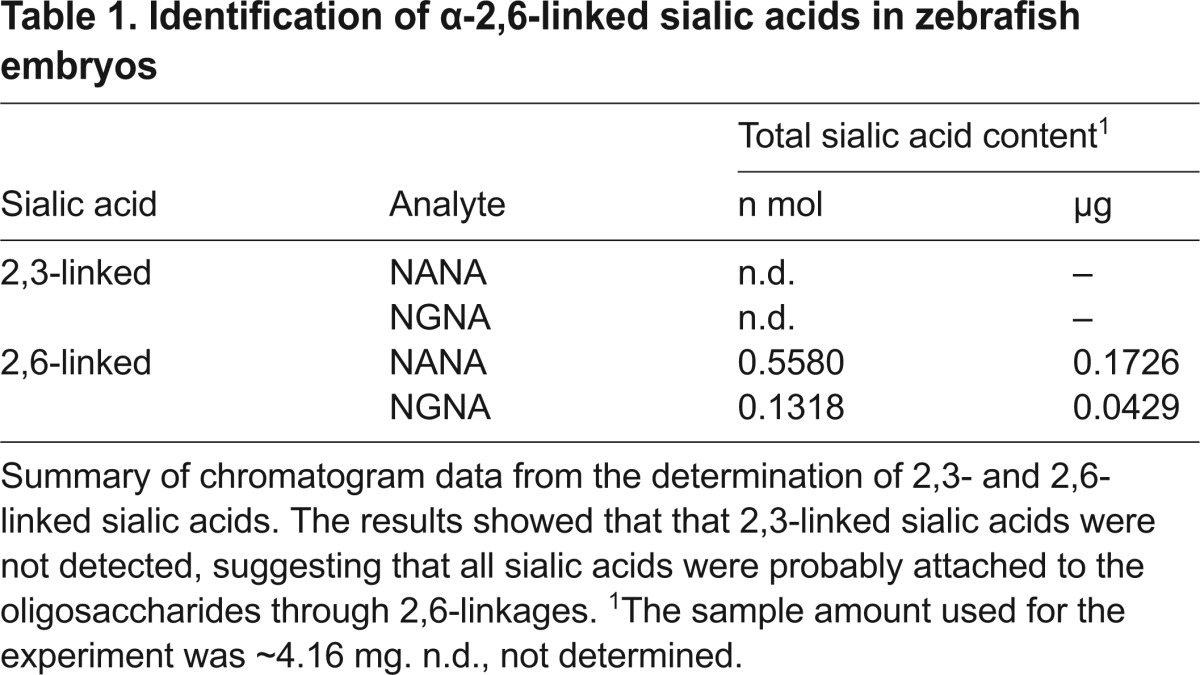
Identification of α-2,6-linked sialic acids in zebrafish embryos

### IAV replicates in and kills infected zebrafish

**Fig. 1.**
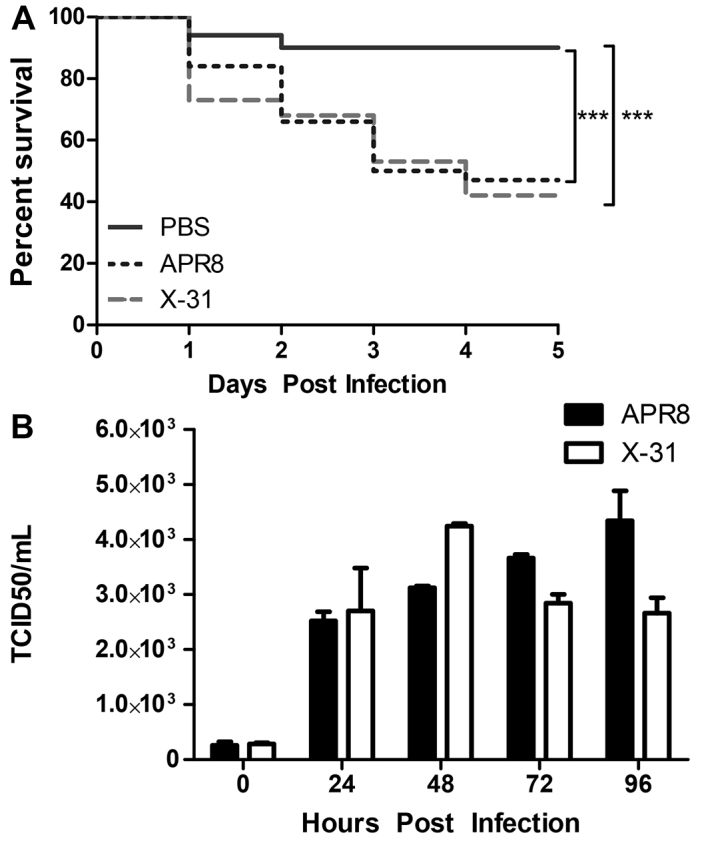
**Zebrafish embryos are susceptible to IAV infection, and IAV replicates in zebrafish hosts.** PBS- or IAV-injected zebrafish embryos were monitored for 5 days post infection for quantification of cumulative percent survival (A) and viral burden (B). The results represent three separate experiments. (A) Cumulative percentage survival of zebrafish infected at 48 hpf with IAV APR8 or X-31 (*n*=50 fish per treatment). Significantly fewer zebrafish survived in either IAV-infected group compared with controls (***P<0.001). (B) Quantification of viral burden in zebrafish infected with APR8 or X-31 (*n*=20 fish per treatment). Viral burden increases 10- to 20-fold in APR8- or X-31-infected zebrafish. In APR8-infected embryos, viral burden increased steadily over time through to 96 hpi. In X-31-infected zebrafish, viral burden peaked around 48 hpi. These data demonstrate IAV replicates within zebrafish and suggest that death in IAV-infected zebrafish is likely to be caused by infection with the virus.

To determine whether human isolates of IAV can cause disease in zebrafish embryos, zebrafish were injected with different strains of influenza virus [APR8 (H1N1) or X-31 (H3N2)] or sterile PBS. Approximately 5×10^3^ units of the 50 percent egg infectious dose (EID_50_) of IAV was injected intravenously at 48 hpf via the Duct of Cuvier (DC) to model influenza viremia. Mortality was monitored over a 5-day period and began at 24 hours post infection (hpi) (Fig. 1A). Embryos injected with APR8 or X-31 showed a cumulative mortality of 54% and 59%, respectively, whereas over 90% of the fish injected with PBS alone survived.

Viral burden was quantified from 24–96 hpi using 50 percent tissue culture infective dose (TCID_50_) assays in order to demonstrate replication of human IAV in zebrafish and to determine whether death in IAV-infected zebrafish correlated with viral burden. Viral burden was determined and revealed that embryos infected with APR8 were initially injected with 2.55×10^2^ TCID_50_/ml, and that this increased to 4.34×10^3^ TCID_50_/ml at 96 hpi. Similarly, embryos infected with X-31 had an initial viral titer of 2.80×10^2^ TCID_50_/ml, which increased to 2.66×10^3^ TCID_50_/ml at 96 hpi (Fig. 1B). The titers of both strains of IAV increased ~10-fold in infected zebrafish over 96 hours. Taken together, these results show that IAV can replicate in zebrafish and cause a systemic infection that leads to mortality in a zebrafish host.

### Antiviral genes are expressed in zebrafish infected with IAV

**Fig. 2.**
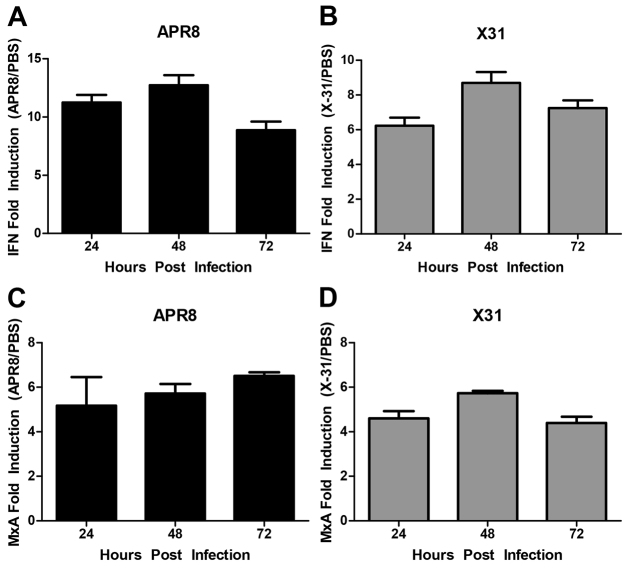
***ifnφ1* and *mxa* mRNA expression is upregulated in IAV infected zebrafish.** Results from qPCR experiments where *ifnφ1* and *mxa* expression is normalized to 18S mRNA. The data presented represent three individual experiments, *n*=10 fish per treatment. Each bar represents the mean fold induction of IAV-infected samples compared with corresponding controls. (A,B) *ifnφ1* transcripts were upregulated 8- to 12-fold in APR8-infected zebrafish, with the peak transcript level occurring at 48 hpi. (C) *mxa* expression was upregulated 5- to 6-fold with expression steadily increasing in APR8-infected zebrafish through to 72 hpi, and (D) peak expression occurred in X-31-infected zebrafish at 48 hpi. These data suggest that antiviral signaling is elicited in IAV-infected zebrafish.

The use of animal models to study influenza infection allows for not only virological assays, but also the reciprocal host response to IAV. A better understanding of the host’s innate antiviral defenses against IAV could lead to new targets for antivirals or adjuvant therapies. Although zebrafish have both innate and adaptive immunity, embryos and larvae rely on the innate immune response for the first 4–6 weeks of development. This temporal separation of innate and adaptive immunity in zebrafish allows for the study of the innate immune response to viral infection independent of adaptive immunity. One of the host’s first innate immune responses to viral attack is the induction of an antiviral state, which limits the spread of infection. Interferon (IFN) is a crucial antiviral signaling molecule and an indicator of the host antiviral state. Induction of IFN also leads to the expression of Mx, a member of the GTPase protein family, which was first recognized for its expression during the antiviral response against IAV (Lindenmann, 1962). Zebrafish IFN and IFN receptors have been previously characterized, and this manuscript uses the IFNΦ nomenclature suggested by the authors (Levraud et al., 2007; Aggad et al., 2009). To determine whether antiviral signaling cascades are induced in a manner similar to that of mammals, zebrafish were infected with IAV through DC injection, and mRNA expression levels of *ifnφ1* and *mxa* were measured in control and IAV-infected zebrafish samples by using quantitative PCR (qPCR) at 24, 48 and 72 hpi. qPCR analyses demonstrated that infection with APR8 caused *ifnφ1* expression to increase within the first 24 hpi, peak at 48 hpi and remain elevated for 72 hpi compared with uninfected controls (Fig. 2A). Zebrafish infected with X-31 exhibited an *ifnφ1* expression profile similar to that of APR8-infected zebrafish (Fig. 2B). These data clearly demonstrate that IAV infection induces *ifnφ1* expression in zebrafish.

Induction of *mxa* expression was also observed in IAV-infected zebrafish. Infection with APR8 resulted in a steady increase in *mxa* expression through to 72 hpi, relative to uninfected controls (Fig. 2C). Infection with X-31 led to increased *mxa* expression that peaked at 48 hpi, but remained elevated through to 72 hpi (Fig. 2D). These results demonstrate that IAV infection also induces *mxa* expression in zebrafish. It has been reported that the *NS1* gene of IAV has the ability to interfere with IFN expression and to function through multiple mechanisms (Hale et al., 2008); however, IFN and downstream targets of IFN signaling are still commonly shown to be induced upon IAV infection (García-Sastre, 2002; García-Sastre, 2011). Taken together, these cytokine profiles show that zebrafish initiate an antiviral state in response to IAV infection through antiviral signaling cascades that are conserved in mammals.

### Systemic IAV infection in zebrafish causes a pathological phenotype

**Fig. 3.**
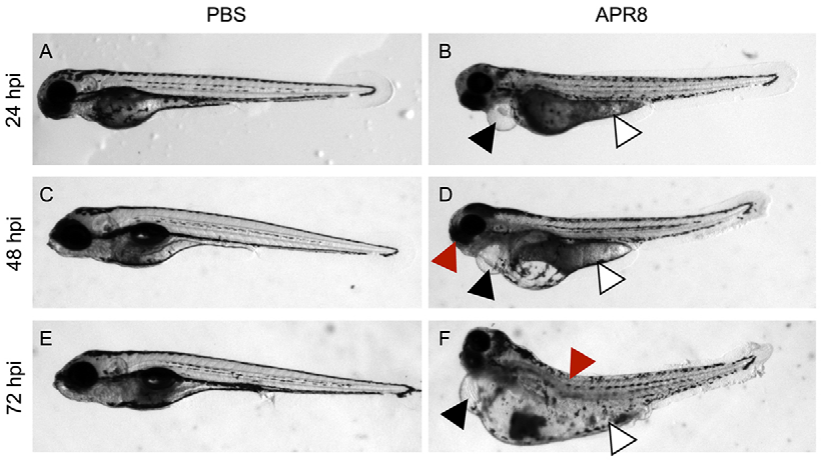
**IAV infection causes a disease phenotype in zebrafish.** Brightfield microscopy of live zebrafish at 24, 48 and 72 hpi, side oriented, anterior left, dorsal top, magnification ×25. The data presented represent three individual experiments, a mean of *n*=7 fish per treatment. (A,C,E) PBS-injected controls. Development proceeded normally in PBS-injected controls at 24 hpi (A), 48 hpi (C) and 72 hpi (E). (B,D,F) APR8-infected zebrafish. Embryos infected with APR8 or X-31 (data not shown) displayed symptoms of infection. IAV-infected zebrafish had pericardial edema (black arrowheads in B,D,F) and yolk sac edema (white arrowheads in B,D,F). IAV-infected zebrafish also had craniofacial abnormalities (red arrowhead in D) and arched backs (red arrowhead in F). The most striking phenotype of IAV-infected zebrafish was generalized edema that worsened over time.

To further characterize the zebrafish innate immune response to IAV viremia, infected embryos were monitored over time for phenotypic and behavioral changes. PBS-injected controls did not display abnormal behaviors or signs of infection over time (Fig. 3A,C,E). However, zebrafish infected with either strain of IAV were lethargic by 24 hpi (data not shown). Edema was commonly observed in the pericardium (Fig. 3B,D,F) and yolk sac (Fig. 3B,D,F) of IAV infected embryos. The edema observed in infected zebrafish worsened over time. Moreover, other pathological phenotypes were observed in IAV-infected zebrafish, including varying degrees of lordotic bending of the spine (Fig. 3F), pigmentation defects, eye abnormalities and craniofacial deformities (Fig. 3D, red arrowhead).

**Fig. 4.**
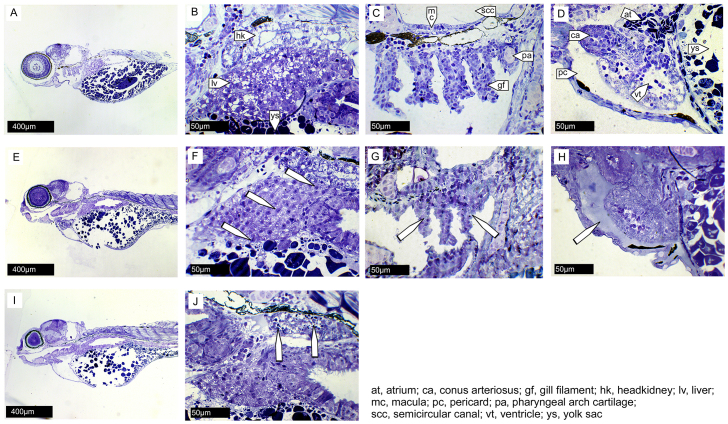
**Histopathology of zebrafish infected with IAV.** Parasagittal semi-thin sections from PBS-, APR8- or X-31-injected zebrafish at 48 hpi, stained with Toluidine Blue (*n*=3 per group, 350 sections per fish). Anterior to the left, dorsal top. PBS control (A-D), APR8-infected (E-H) and X-31-infected (I,J) zebrafish. (B) Head kidney (hk), liver (lv) and yolk sac (ys) of a control zebrafish. No degeneration of cells was observed. (C) Gill and pharyngeal arches adjoined to the occipital region of a control zebrafish. All tissues are without pathological anomalies: semicircular canal of the inner ear (scc) with macula (mc), cartilage of fifth pharyngeal arch (pa) and anterior located gill filaments (gf). (D) The same control fish with healthy heart in front of the yolk sac, atrium (at), ventricle (vt), conus ateriosus (ca) and pericardial volume (pc). (F) The liver of APR8-infected zebrafish. The white arrowheads indicate the presence of degenerative liver tissue, indicated by the necrotic disintegration of nuclei. (G) Gill chamber of APR8-infected zebrafish. The white arrowheads indicate necrotic gill filaments that contain many vacuolated cells. (H) Heart region of APR8-infected zebrafish. The white arrowhead indicates the fluid-filled pericardium. The histological analysis confirms the edema seen in IAV-infected zebrafish (Fig. 3B,D,F). (J) The head kidney of X-31-infected zebrafish shows clear signs of necrotic tissue degeneration. The white arrowheads indicate the presence of degenerating cells in the hematopoietic part of the head kidney.

For additional investigation of pathophysiology, histopathology sections were prepared from zebrafish with IAV viremia at 48 hpi. Histopathological analyses confirmed the presence of clinical symptoms of influenza, including necrotic tissues and edema (Taubenberger and Morens, 2008). Neither tissue degeneration nor edema was apparent in PBS-injected zebrafish (Fig. 4A–D). In APR8-infected fish, necrotic degeneration of liver and gill tissues was observed (Fig. 4F,G). Necrotic cells in the hematopoietic tissue of the head kidney were evident in X-31-infected fish (Fig. 4J). Histopathology confirmed that pericardial edema occurred in APR8-infected zebrafish (Fig. 4H) and not in PBS-injected fish (Fig. 4D). Foci of accumulated granulocytes or monocytes in virus-infected fish were not observed, suggesting widespread dissemination of the virus and a lack of localized secondary bacterial infection. Taken together, these pathology analyses of IAV-infected zebrafish show edema and tissue destruction, which is consistent with a primary systemic influenza infection with multiple organ involvement.

### *In vivo* imaging reveals infection dynamics

**Fig. 5.**
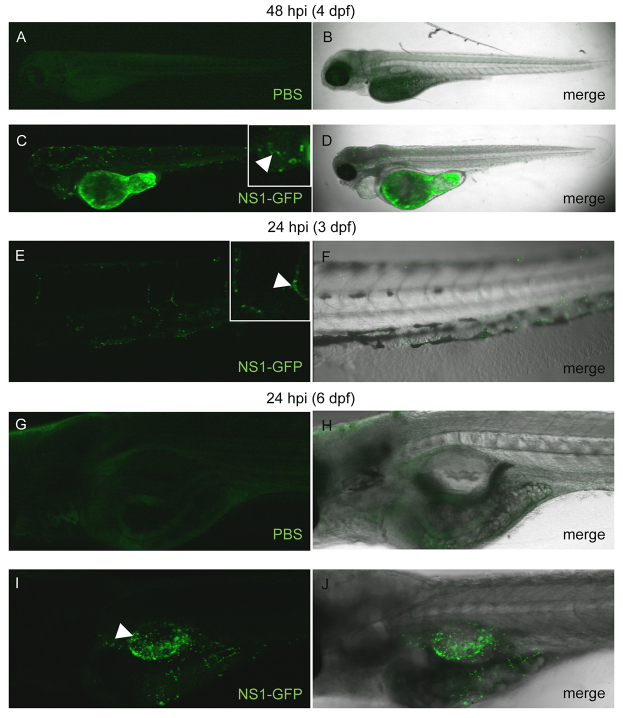
**NS1-GFP shows infection of the zebrafish cardiovascular system and swimbladder.** (A–D) Single focal planes of PBS- or NS1-GFP-injected, fixed casper mutant zebrafish at 48 hpi, side mounted, anterior left, dorsal top, ×4 magnification. (A) Fluorescence micrograph of a PBS-injected control showing background autofluorescence. The data presented represent three individual experiments, a mean of *n*=6 fish per treatment. (B) Panel of PBS control showing merged fluorescence and brightfield micrographs. (C) Fluorescence micrograph of NS1-GFP-infected zebrafish. Punctate fluorescence confirms viral replication. Inset is a computer magnification of the pericardial region. White arrowhead points to fluorescence in the heart. (D) Merge of NS1-GFP-infected embryo fluorescence and brightfield micrographs. (E,F) 3D reconstruction of NS1-GFP-infected, live zebrafish, 24 hpi, side mounted, anterior left, dorsal top, magnification ×10. Punctate fluorescence was observed in major blood vessels. Inset is a computer zoom of two infected blood vessels. White arrowhead denotes fluorescence in an intersomitic blood vessel. (G–J) 3D reconstruction of casper mutant zebrafish after injection of either PBS or NS1-GFP into the swim bladder at 5 dpf, fixed at 24 hpi, side mounted, anterior left, dorsal top. (G) Fluorescence micrograph of zebrafish with PBS injected into the swim bladder, the image shows background autofluorescence. The data represent two individual experiments, a mean of *n*=5 fish per treatment. (H) Merged panel of PBS-injected control fluorescence and brightfield micrographs. (I) Fluorescence micrograph of NS1-GFP swim bladder infection. Green punctate fluorescence is likely to show infection of epithelial cells around the swim bladder. (J) Fluorescence and brightfield micrograph merge for NS1-GFP swim bladder infection. Infection with the NS1-GFP reporter virus in transparent zebrafish larva allows for visualization of viral replication, spread, resolution of infection and cell tropism *in vivo*.

In addition to the isolation of the innate immune response in zebrafish embryos and larvae, early-life stage zebrafish are also optically transparent and can be used for *in vivo* microscopy studies. A genetically modified strain of IAV has been engineered previously, in which green fluorescent protein (GFP) is fused to the NS1 segment of APR8 (named NS1-GFP) (Manicassamy et al., 2010). GFP is therefore not incorporated into IAV virus particles, and thus GFP expression reflects viral infection and replication because GFP will only be expressed in the infected cells in which NS1 is translated. To visualize IAV infection dynamics and cell tropism *in vivo*, NS1-GFP was injected into the DC of 2-dpf zebrafish. Infection dynamics were monitored over time through confocal fluorescence microscopy. At 48 hpi, the time at which histopathology analysis was performed, NS1-GFP infection caused mild heart edema and fluorescence was observed in the yolk sac, heart and other locations throughout the head, trunk and tail (Fig. 5C,D). Zebrafish injected with PBS exhibited background levels of autofluorescence, but did not recapitulate the punctate fluorescence pattern seen in NS1-GFP-infected zebrafish (compare Fig. 5A,B with Fig. 5C,D). DC injection of NS1-GFP most consistently resulted in fluorescence in cells of the cardiovascular system, including the heart (Fig. 5C and inset) and blood vessels (Fig. 5E and inset). The fluorescence pattern observed was reminiscent of *fli1*-promoter-driven expression (endothelium specific) (Lawson and Weinstein, 2002), suggesting that DC injection of IAV results in infection of vascular endothelial cells in zebrafish. IAV is known to infect cultured human lung microvascular endothelial cells (Armstrong et al., 2012), thus supporting that IAV cell tropism might be conserved between zebrafish and humans.

In humans, IAV infection can lead to viremia, but IAV more commonly infects epithelial cells of the upper and lower respiratory tract. Zebrafish do not possess the equivalent of a mammalian lung, but the swimbladder is considered to be an anatomically and functionally analogous organ (Robertson et al., 2007; Winata et al., 2009; Zheng et al., 2011). A swimbladder infection model has recently been developed for the fungal pathogen *Candida albicans*, introduced through immersion (Gratacap et al., 2013) and by injection (Brothers et al., 2013). Hence, we attempted to model a localized, epithelial IAV infection in zebrafish by injecting IAV directly into the swimbladder subsequent to its inflation (5 dpf). Injection of NS1-GFP into the swimbladder resulted in dense, punctate fluorescence in this region at 24 hpi (Fig. 5I,J), which was not observed in PBS-injected fish (Fig. 5G,H). The ability to visualize this fluorescent reporter strain of IAV *in vivo* highlights one of the advantages of using the zebrafish model to study infectious diseases and suggests that IAV infects cells that line surfaces of the body (i.e. epithelia and endothelia) in zebrafish as it does in humans.

### The neuraminidase inhibitor Zanamivir confers protection against IAV infection in zebrafish

**Fig. 6.**
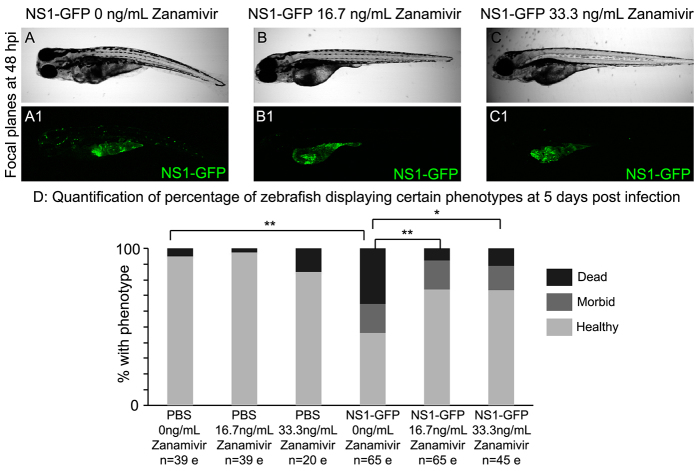
**Antiviral treatment with Zanamivir confers protection after infection in zebrafish.** Zanamivir (16.7 ng/ml or 33.3 ng/ml) was administered at 3 hpi, and Zanamivir-containing media was changed twice daily. (A–C1) Single focal planes of NS1-GFP-injected, live zebrafish at 48 hpi, side mounted, anterior left, dorsal top, ×4 magnification. Lettered panels are brightfield images, and numbered panels are fluorescence micrographs. (A,A1) NS1-GFP-infected zebrafish. Note fluorescence in the yolk region, as well as punctate fluorescence throughout the body. (B,B1) NS1-GFP-infected zebrafish treated with 16.7 ng/ml Zanamivir. (C,C1) NS1-GFP-infected zebrafish treated with 33.3 ng/ml Zanamivir. Note that treatment with Zanamivir at either dose drastically reduced the punctate fluorescence in the body of the zebrafish, whereas fluorescence in the yolk region remained despite antiviral treatment. The data presented represent three individual experiments, a mean of *n*=20 fish per treatment. (D) Quantification of healthy, morbid and dead zebrafish at 5 days post infection with NS1-GFP. These data are compiled from three independent experiments, and the number of zebrafish per treatment group is labeled on the graph. e, embryos. Fisher’s exact test two-tailed *P*-values (with Bonferroni correction) were used to determine whether the proportion of dead versus alive (healthy and morbid) zebrafish significantly differed between treatments. Addition of either dose of Zanamivir to PBS-injected controls did not significantly affect the proportion of zebrafish that died. Infection with NS1-GFP significantly increased the proportion of dead zebrafish compared with the PBS-injected group (*P*=0.0018). Addition of either dose of Zanamivir significantly decreased the proportion of NS1-GFP-infected zebrafish that died compared with the infected, untreated group (*P*=0.0012 and *P*=0.0246 for 16.7 ng/ml and 33.3 ng/ml Zanamivir, respectively). *P<0.05, **P<0.01.

A transparent host susceptible to IAV infection, together with the NS1-GFP strain of IAV (Manicassamy et al., 2010), affords us the unique opportunity to acquire *in vivo* data about potential test compounds that could disrupt infection progression. Zebrafish have been utilized for many high-throughput drug screens (Taylor et al., 2010), and because IAV rapidly mutates and becomes resistant to antivirals, a zebrafish model of human IAV infection will be invaluable for testing the antiviral potential of chemical compounds. As a proof-of-concept experiment, the antiviral Zanamivir was administered to infected zebrafish in order to test whether it could alter IAV infection. Zanamivir is a neuraminidase inhibitor that disrupts viral exit from infected cells and has known antiviral activity against influenza A and B viruses. Zanamivir was administered through immersion at clinically relevant doses (16.7 or 33.3 ng/ml) twice daily, beginning at 3 hpi (when GFP expression is first observed near to the injection site in NS1-GFP-infected zebrafish). GFP expression was monitored and documented by using confocal microscopy, and both behavior and gross pathology were assessed. Treatment with either dose of Zanamivir did not alter behavior or the overall phenotype in PBS-injected controls (data not shown). NS1-GFP-infected zebrafish that had been treated with either dose of Zanamivir were more mobile, more likely to undergo the normal transition from a side-laying to upright orientation and displayed reduced pericardial and yolk sac edema compared with untreated infected zebrafish (data not shown, compare Fig. 6B,C with Fig. 6A). Confocal imaging of GFP expression in NS1-GFP infected zebrafish with and without Zanamivir treatment revealed that both doses of Zanamivir reduced GFP expression in the body of larval zebrafish (Fig. 6B1,C1) compared with infected untreated zebrafish (Fig. 6A1), but did not visibly inhibit GFP expression in the yolk region (Fig. 6A1–C1). Although the mortality of PBS-injected zebrafish was not significantly altered by either dose of Zanamivir, NS1-GFP infection significantly increased mortality compared with PBS-injected groups. Both doses of Zanamivir significantly rescued the mortality of NS1-GFP-infected zebrafish (Fig. 6D). The results of these experiments demonstrate that a compound with established antiviral activity in humans has similar activity in zebrafish and reinforce the validity of utilizing this model for chemical screens to identify novel anti-influenza compounds.

## DISCUSSION

Influenza infections are a threat to human and animal health, as well as to the global economy. Despite annual influenza vaccines and decades of research, influenza still causes substantial morbidity and mortality worldwide. Influenza research using animal models is crucial for the introduction of new antiviral drugs that are effective against viruses resistant to current antiviral treatments. These models also enhance our understanding of the host signaling pathways and defense systems that alleviate or exacerbate disease. Here, the first studies using zebrafish as a model system for human IAV research are presented. Infection of zebrafish embryos with IAV is demonstrated, and the subsequent antiviral signaling and pathology are characterized. Injection of IAV into the bloodstream results in increased viral burden and mortality compared with uninfected controls. Cytokine profiles show that antiviral signaling is induced in IAV-infected zebrafish. Gross pathology and histopathology reveal typical symptoms of influenza. Infection with a GFP reporter strain of IAV enables visualization of infected cells *in vivo*, and GFP expression and mortality can be ameliorated by antiviral treatment, supporting the relevance of this model for antiviral drug screening. Owing to the optical clarity of zebrafish embryos and their potential for high-throughput genetic and drug screening, this zebrafish embryo model of IAV infection will be an important resource for dissecting molecular mechanisms of host-pathogen interactions *in vivo*, as well as for identifying new antiviral therapies.

### Implications for the presence of α-2,6-linked sialic acids in zebrafish embryos

Certain characteristics of the zebrafish model system make it an attractive option for infectious disease research. To date, no naturally occurring viral infections of zebrafish have been reported (Crim and Riley, 2012). It has been shown that zebrafish can be infected with many fish-specific viruses (LaPatra et al., 2000; Sanders et al., 2003; Phelan et al., 2005; Novoa et al., 2006; Lu et al., 2008; Xu et al., 2008; Encinas et al., 2010), and there may be an untapped potential for zebrafish to host human viral pathogen infections. One objective of this study was to determine whether zebrafish embryos could serve as a host for infection with mammalian and/or human isolates of IAV. A necessary prerequisite for human IAV infection is the presence of α-2,6-linked sialic acid modifications on host glycolipids or glycoproteins. IAV hemagglutinin spikes bind to host sialic acids, and viruses enter host cells through receptor-mediated endocytosis. Because human IAV preferentially binds to α-2,6-linked sialic acids, an analysis of the sialic acid linkage types present in zebrafish embryos was conducted. High performance anion exchange chromatography with pulsed amperometric detection (HPAEC-PAD) analysis demonstrated that α-2,6-linked sialic acids are present in 2-dpf zebrafish embryos. Terminally linked sialic acids can occur in α-2,3, α-2,6, or α-2,8 linkages. α-2,6- but not α-2,3-linked sialic acids were detected in zebrafish embryos at 2 dpf. We did not attempt to detect α-2,8-linked sialic acids in our sample; however, others have previously demonstrated that zebrafish synthesize a variety of sialylated glycoconjugates during the early stages of development (Marx et al., 2001; Chang et al., 2010; Dehnert et al., 2012; Langhauser et al., 2012; Schaper et al., 2012). Identification of the sialic acid linkages present at different developmental stages, including juvenile and adult zebrafish, will further inform which human viral diseases could potentially be studied using the zebrafish model. The presence of α-2,6-linked sialic acids in zebrafish embryos suggests that this model organism might be able to support infection with other pathogens that also bind to α-2,6-linked sialic acids. Examples of additional pathogens that bind to α-2,6-linked sialic acids include certain members of the *Coronaviridae*, *Paramyxoviridae*, *Caliciviridae*, *Picornaviridae*, *Reoviridae*, *Polyomaviridae*, *Adenoviridae* and *Parvoviridae* virus families (Neu et al., 2011; Matrosovich et al., 2013).

In addition to requirements for certain cellular receptors, another important factor to consider in zebrafish infection studies is the optimal incubation temperature for viruses. Zebrafish can be grown in a temperature range within that of the human respiratory tract (from 25 to 33°C). Thus, zebrafish might be well suited to modeling human respiratory viral infections, especially given the similarities between the zebrafish swimbladder and the human lung (Robertson et al., 2007; Winata et al., 2009; Zheng et al., 2011; Gratacap et al., 2013), and recent demonstrations of infection of the zebrafish swimbladder with a human fungal pathogen (Gratacap et al., 2013).

### Advantages to using zebrafish for infection studies

Modeling infections in zebrafish of all life stages has added insights into host-pathogen interactions *in vivo* (Dooley and Zon, 2000; Trede et al., 2004; Sullivan and Kim, 2008; Kanther and Rawls, 2010). Some pathogens can be transmitted to zebrafish embryos or adults through immersion, whereas others must be injected. Immersion of zebrafish embryos in egg media containing 7×10^6^ EID_50_ IAV did not result in infection (data not shown). We have not yet attempted to immerse or inject adult zebrafish with IAV. The zebrafish embryo infectious disease model has certain advantages that will be valuable for IAV studies, including temporal segregation of innate and adaptive immunity, amenability to genetic manipulation, optical transparency and the potential for high-throughput chemical screens. Some of these advantageous characteristics carry over into adult zebrafish, and the study of IAV infection in adult zebrafish is likely to provide additional unique experimental opportunities and insights into this infectious disease.

During zebrafish development, functionality of the innate and adaptive arms of the immune response is temporally separated. The innate immune response is active as early as 24 hpf; however, the adaptive immune response is not fully functional during the first 4–6 weeks of zebrafish development (Lam et al., 2004; Kanther and Rawls, 2010). Therefore, zebrafish embryos provide the possibility to study the role of innate immunity during IAV infection with little to no interference from the adaptive immune response. The transparency of zebrafish embryos coupled with the isolation of innate immunity provides the unique opportunity, not possible in any other vertebrate animal models of IAV infection, to observe spatiotemporal dynamics of IAV infection and the innate immune response *in vivo*. Infection with a fluorescent reporter strain of IAV in a transparent host will be useful for *in vivo* IAV cell tropism studies and to understand in greater detail the progression of influenza in response to experimental manipulations. The use of fluorescent IAV and fluorophore-labeled transgenic zebrafish lines that enable direct visualization of phagocytes (Mathias et al., 2006; Renshaw et al., 2006; Hall et al., 2007; Ellett et al., 2011), antiviral signaling and/or proinflammatory signaling will allow for visualization of interactions between pathogens and the innate immune system in real time. Mutant lines and morpholino-mediated protein knockdown provide opportunities to manipulate the host response and to determine roles for gene products in the innate immune response to IAV. Leveraging the advantages of zebrafish embryos to isolate and dissect roles for innate immunity in the host response to IAV infection and disease severity could be particularly informative in light of the recent findings implicating dysregulated innate immunity in mortalities resulting from IAV infection in mammals (Brandes et al., 2013). Because the innate immune response is also known to promote adaptive immunity, a better understanding of the innate immune response to IAV infection could potentially both reduce mortality and improve the success of current vaccination and preventative strategies.

As zebrafish display adaptive immunity later in their development, the model presented here could possibly be extended to study the adaptive immune response and vaccine efficacy against IAV infection. Unlike embryonic zebrafish, adult fish are not optically transparent, and genetic manipulation is currently more challenging in adults than in embryos. However, pigmentation mutant lines of zebrafish (e.g. casper) (White et al., 2008) could potentially allow for live imaging of fluorescent host-pathogen interactions in adult zebrafish. Additionally, large-scale mutagenesis projects and targeted mutagenesis technologies, such as zinc finger nucleases (ZFN) (Meng et al., 2008), transcription activator-like effector nucleases (TALEN) (Huang et al., 2011) and clustered regularly interspaced short palindromic repeats (CRISPR) (Hwang et al., 2013), are generating many new zebrafish mutant lines. These mutant lines could then be used to assess gene function in the host response to IAV infection and vaccine success in older zebrafish.

The ability to conduct high-throughput drug screens in embryos, and even in adult zebrafish, is a major advantage to the use of this vertebrate animal model for infection studies. IAV is rapidly developing resistance to the few anti-influenza compounds available. We have laid the foundation for use of the zebrafish embryo infection model to screen for new anti-influenza drugs by demonstrating that a fluorescent reporter strain of IAV infects zebrafish and that NS1-GFP expression (i.e. viral replication and infection) in zebrafish responds to a known anti-influenza compound as it does in humans. Fluorescence in NS1-GFP-infected zebrafish could be used to quantify viral burden or follow the progression of IAV infection *in vivo* in response to a range of test compounds. When using animal models to screen for potential therapeutic molecules, it is important to balance maximal protection with minimal side effects and to consider clinically relevant doses. This can be accomplished in zebrafish through dose-response titration experiments and comparison with clinically relevant dosages. The antiviral Zanamivir is administered to humans through intravenous or oral inhalation routes. Serum concentrations of Zanamivir were calculated in healthy human volunteers following twice daily treatment with Zanamivir at multiple doses used in the clinic (100, 200 or 600 mg intravenous solution or 10 mg oral inhaled) and the maximum serum Zanamivir concentrations reported were between 9.83 and 45.3 ng/ml (Shelton et al., 2011). Assuming an average of 6 litres of human blood volume, 100 or 200 mg doses of Zanamivir given intravenously would result in ~16.7 or 33.3 ng/ml Zanamivir in the blood. We mimicked this dose of Zanamivir in our zebrafish influenza infection model by immersing zebrafish in 16.7 or 33.3 ng/ml Zanamivir solutions and changing the media twice daily. Both doses of Zanamivir significantly improved the survival of NS1-GFP-infected zebrafish and reduced the expression of GFP in the body of the zebrafish without reducing expression of GFP in the yolk region. The zebrafish yolk is encased by a layer of cells that share cytoplasm, called the yolk syncytial layer. The yolk syncytial layer is in close proximity to the initial site of NS1-GFP infection, the DC. Therefore, although Zanamivir administration at 3 hpi can largely prevent NS1-GFP infection in the body of the zebrafish, it cannot prevent local infection in the yolk syncytial layer. This proof-of-concept experiment suggests the exciting possibility that the zebrafish embryo model of IAV infection can be used to rapidly identify novel anti-influenza drugs that might ultimately translate into human therapies.

### Conclusion

The results of the present study further establish the zebrafish as a valuable animal model for studying host-pathogen interactions. This zebrafish model of human IAV infection has great potential for the exploration of the relationships between viral and host factors that contribute to the pathogenesis of influenza. Some of these factors could become targets for the development of new antiviral interventions or adjuvant therapies. A zebrafish model of IAV infection will provide a powerful new tool in the search for new ways to prevent and treat influenza.

## MATERIALS AND METHODS

### Zebrafish care and maintenance

Zebrafish (*Danio rerio*) used in this study were handled in accordance with the recommendations in the Guide for the Care and Use of Laboratory Animals of the National Institutes of Health. The protocols used in this study were approved by the Institutional Animal Care and Use Committee (IACUC) at the University of Maine (Protocol Number: A2013-06-03). Zebrafish were maintained in the Zebrafish Facility at the University of Maine, Orono. The facility was maintained according to IACUC standards. IACUC approved guidelines for zebrafish care followed the standard procedures (www.zfin.org) of a 14-hour light, 10-hour dark cycle at 28°C. Embryos were obtained by natural spawnings of adult AB or casper mutant zebrafish. Fertilized eggs were collected and raised in egg water (60 μg/ml Instant Ocean sea salts; Aquarium Systems, Mentor, OH) at 28°C.

### Determination of total sialic acids

Analysis of the total sialic acid was performed at the Complex Carbohydrate Research Center of the University of Georgia. Zebrafish embryos were stored in 70% ethanol, constituting the sample matrix. The ethanol from the sample matrix was first evaporated from each tube under a stream of nitrogen gas, which was subsequently combined into one tube. The volume was further reduced to between 1.0 and 1.5 ml under a stream of nitrogen gas and then precipitated with cold acetone:water (crude protein extract). Precipitation of the sample was performed to remove contaminants and extraneous material of the zebrafish. A weighed amount (3.6 mg) of the dried crude protein extract from zebrafish embryos was hydrolyzed with 2 M acetic acid to liberate sialic acids from glycoproteins. After hydrolysis, the sample was dried under a stream of air, re-dissolved in cold water, sonicated for 5 minutes and then transferred into an injection vial. A mixture of standards for sialic acids at known concentrations was subjected to hydrolysis conditions under identical conditions to those used for the samples.

The sialic acids were analyzed by HPAEC-PAD using a Dionex ICS3000 system equipped with a gradient pump, an electrochemical detector and an autosampler. The individual sialic acids were separated by a Dionex CarboPac PA20 (3×150 mm) analytical column with an amino trap. The gradient program used the following mobile phase eluents: 100 mM NaOH, and 1M sodium acetate in 100 mM NaOH. Injections were made every 35 minutes. All methods were based on protocols described previously by Hardy and Townsend (Hardy and Townsend, 1994). Four concentrations of the standard mixture were prepared by serial dilution to establish a calibration equation. The number of moles of each residue in the sample was quantified by linear interpolation from the calibration equation.

### Determination of α-2,3-and -2,6-linked sialic acids

A weighed amount (~4.16 mg) of the dried crude protein extract from zebrafish embryos was prepared for an experiment to determine and quantify α-2,3- and -2,6-linked sialic acids. The sample was first digested with trypsin and chymotrypsin for 24 hours to release glycopeptides. The digest was passed through a C18 Sep-Pak Vac 1cc (50 mg) cartridge (Waters Corporation, Milford, MA) for purification, and the glycopeptides were eluted and lyophilized. The glycopeptides were subsequently treated with sialidase specific to cleavage of α-2,3-linked sialic acids. The digest was passed through a C18 Sep-Pak Vac 1 cc (50 mg) cartridge (Waters Corporation, Milford, MA) and α-2,3-linked sialic acids were eluted with 5% acetic acid, and remainder glycopeptides were eluted into a separate tube, lyophilized and finally treated with general neuraminidase to release α-2,6-linked and all remaining sialic acids from the glycoprotein.

A mixture of standards for sialic acids at known concentrations was prepared and not hydrolyzed at the same time as the samples were treated. The sialic acids in the sample and in the standard mixture were analyzed by HPAEC-PAD as described above. Four concentrations of the standard mixture were prepared by serial dilution to establish a calibration equation. The number of moles of each residue in the sample was quantified by linear interpolation from the calibration equation.

### Microinjection of IAV

Embryos were manually dechorionated at 48 hpf with fine forceps (DuMont no. 5). Prior to injections, fish were anesthetized in tricaine solution and lined up on a 3% agarose gel in a Petri dish before being injected into the DC with 1.5 nl (~5×10^3^ EID_50_) of APR8 or X-31 IAV or 4 nl [~6×10^2^ plaque forming units (PFU) per embryo] of NS1-GFP in PBS with a final concentration of 0.25% phenol red. Dechorionated 5-dpf zebrafish with inflated swimbladders were anesthetized, and fish were injected as above with NS1-GFP in the posterior part of the swimbladder. Sterile PBS including 0.25% phenol red was injected into the DC or swimbladder of control zebrafish. Following injection, zebrafish were maintained at 33°C and the egg water was changed daily. Microinjection was controlled by an MPPI-2 pressure microinjector (Applied Scientific Instruments) and pulled microcapillary pipettes (Sutter Instruments, Novato, CA) were used to inject the virus or PBS.

### Cell culture

Madin-Darby canine kidney (MDCK) cells were purchased from ATCC (CCL-34) and cultured at 37°C under 5% CO_2_. MDCK cells were maintained in Dulbecco’s modified Eagle’s medium (DMEM) supplemented with 10% heat-inactivated fetal bovine serum, 1% antibiotics (100 units/ml penicillin and 100 μg/ml streptomycin) and 1% L-glutamine (2 mM). All cell culture media was purchased from GIBCO Life Technologies.

### Virus, mortality curves and viral burden assays

The influenza viral strains H1N1 (APR8/34, part no. 490710) and H3N2 (X-31 A/Aichi/68, part no. 490715) were purchased from Charles River Laboratories (North Franklin, CT). The virus strains were stored at −80°C, thawed at room temperature and diluted in sterile PBS to the desired infectious dose.

Wild-type zebrafish embryos were injected and maintained as described above. For mortality experiments, water was changed daily and mortality was monitored and counted from 24–96 hpi.

Viral titer of influenza virus in zebrafish was quantified using a TCID_50_ end point dilution assay. Viral burden assays were performed in MDCK cells. For viral burden assays, 20 fish were collected immediately following injection (0 hpi) and then between 24 and 96 hpi at 24-hour intervals. Water was changed daily and dead fish were removed prior to collection at each time point. Embryos were homogenized in DMEM (GIBCO Life Technologies, Carlsbad, CA) supplemented with 25 mM HEPES, 0.2% BSA, 100 units/ml penicillin and 100 μg/ml streptomycin. Homogenates were centrifuged at 15,000 ***g*** at 4°C for 10 minutes. Supernatants were removed in two aliquots of 105 μl and frozen at −80°C before the TCID_50_ assay.

MDCK cells were plated 24 hours prior to the assay in 96-well plates (Corning Incorporated, Corning, NY) such that the wells were ~70% confluent at the time of virus addition. For MDCK cells, 100 μl of cell suspension (~15,000 cells) was added to each well of a 96-well plate. At the time of the assay, virus-containing supernatants were thawed and immediately serially diluted in dilution media (unsupplemented Eagle’s minimal essential medium for MDCK cells) prior to use in TCID_50_ assays. Cells were overlaid with 100 μl of virus dilution per well and incubated for 2 hours at 37°C under 5% CO_2_. Immediately following the 2-hour incubation period, an additional 100 μl of dilution media was added, and plates were covered with adhesive sealing film (Excel Scientific, Victorville, CA) and returned to 37°C under 5% CO_2_. For all assays, uninfected wells were maintained as controls. Cytopathic effects (CPE) were observed after 3 days. TCID_50_/ml was calculated according to the Reed-Muench method. Each bar in the graph shows a representative experiment of TCID_50_/ml values. Three independent experiments were performed. Error bars were calculated as s.e.m.

### Zanamivir treatment

Zanamivir (AK Scientific, Union City, CA) was dissolved in nuclease-free water to 10 mg/ml, aliquoted and frozen at −20°C. NS1-GFP-and PBS-injected zebrafish were kept in 60-mm Petri dishes at a density of 20–25 zebrafish per dish in 10 ml of egg water at 33°C. At 3 hpi, egg water was removed and replaced with egg water containing 0, 16.7 or 33.3 ng/ml Zanamivir. Egg water with the desired concentrations of Zanamivir was remade and replaced twice daily for 5 days post infection.

### RNA extraction, cDNA synthesis and real-time PCR

Total RNA was extracted from whole embryos at 24, 48 and 72 hpi by homogenizing 10 fish at each time point, treating with TRIzol reagent (Invitrogen, Carlsbad, CA) and subsequently storing at −80°C. RNA was extracted according to the manufacturer’s protocol. Reverse transcription reactions were performed in a manner similar to those reported previously (Phelan et al., 2005) to synthesize cDNA using Bio-Rad iScript (Bio-Rad Laboratories, Hercules, CA). Quantitation of *ifnφ1* (Aggad et al., 2009) and *mxa* was performed through real-time PCR using an I-cycler IQDetection System (Bio-Rad Laboratories, Hercules, CA). The cycling parameters used were chosen as described previously (Phelan et al., 2005). Fluorescence measurements were made at each cycle during the annealing step, and the copy number was determined based on a standard curve using the iCycler software. The value for each sample was normalized to the corresponding 18S value to determine relative copy number.

### Histopathology

For the histopathological examination, zebrafish were fixed with a mixture of 1.5% glutaraldehyde and 1.5% paraformaldehyde in 0.1 M sodium cacodylate (pH 7.4) buffer containing 0.001% CaCl_2_, they were subsequently rinsed in 0.1 M sodium cacodylate buffer containing 10% sucrose. Postfixation staining of membrane lipids was performed in 1% OsO_4_ that had been dissolved in 0.1 M sodium cacodylate buffer containing 8% sucrose, staining was performed for 2 hours at room temperature. Subsequently, specimens were embedded in Epon epoxy resin. Specimens of all experimental groups were serial semi-thin cross-sectioned and serial semi-thin parasagittal sectioned (1 μm, Reichert Ultracut microtome), stained with Toluidine Blue and mounted in DPX.

### Imaging

A Zeiss stereomicroscope running Axiovision software was used to obtain the time course of gross pathology images. A Zeiss Imager-Z compound microscope and Axiovision software was used for the histopathological analysis. An Olympus Fluoview IX-81 inverted microscope with FV1000 confocal system was used for fluorescence and brightfield imaging of PBS-injected or NS1-GFP-infected embryos. Images were obtained using the ×4 [0.16 numerical aperture (NA), Olympus] or ×10 (0.4 NA, Olympus) objectives.

## Supplementary Material

Supplementary Material
