# Supplementary material for: Influenza A virus infection in zebrafish recapitulates mammalian infection and sensitivity to anti-influenza drug treatment
Source: Dis Model Mech. 2014 Sep 4;7(11):1227–37. doi: 10.1242/dmm.014746 (PMC4213727; doi:10.1242/dmm.014746)
Supplement: Supplementary Material [file supp_7_11_1227__index.html]

Influenza A virus infection in zebrafish recapitulates mammalian infection and sensitivity to anti-influenza drug treatment — Supplementary Material 

# Influenza A virus infection in zebrafish recapitulates mammalian infection and sensitivity to anti-influenza drug treatment

## DMM014746 Supplementary Material

**Files in this Data Supplement:**

- **Supplementary Material**
