# Supplementary material for: Influenza A virus infection in zebrafish recapitulates mammalian infection and sensitivity to anti-influenza drug treatment
Source: Dis Model Mech. 2014 Sep 4;7(11):1227–37. doi: 10.1242/dmm.014746 (PMC4213727; doi:10.1242/dmm.014746)
Supplement: Supplementary Material [file supp_7.11.1227_DMM014746.pdf]

SUPPLEMENTARY MATERIAL

Fig. S1. Identification of α-2,6-linked sialic acids in zebrafish embryos. NANA- and NGNA-labeled peaks on the α-2,6-linked sialic acid-specific chromatogram from zebrafish embryos reveal the presence of α-2,6-linked sialic acids. This result suggests zebrafish embryos may be able to support infection by human isolates of IAV.

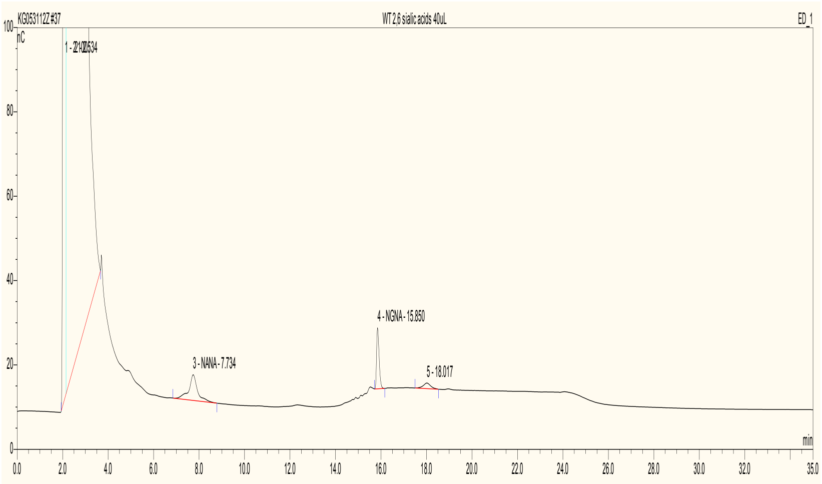

Table S1. Sialic acids composition analysis of WT zebrafish. The total contents of sialic acids in wild-type zebrafish embryos are shown. Both N-acetylneuraminic acid and N-glycolylneuraminic acid were detected from the sample.

		**Total sialic acids content[1]**	
**Sample ID**	**Analyte**	**nanomoles**	**micrograms**
**WT zebrafish embryos**	NANA	6.4359	1.9905
	NGNA	2.8461	0.9258

[1]Sample amount hydrolyzed with 2M acetic acid was ~3.6 mg.
